# Simple theoretical analysis of the photoemission from quantum confined effective mass superlattices of optoelectronic materials

**DOI:** 10.3762/bjnano.2.40

**Published:** 2011-07-06

**Authors:** Debashis De, Sitangshu Bhattacharya, S M Adhikari, A Kumar, P K Bose, K P Ghatak

**Affiliations:** 1 School of Physics, University of Western Australia, The University of Western Australia, 35 Stirling Highway Crawley, Perth, Western Australia 6009; 2 Department of Computer Science and Engineering, West Bengal University of Technology, Kolkata 700074, India; 3 Nanoscale Device Research Laboratory, Centre for Electronics Design and Technology, Indian Institute of Science, Bangalore 560012, India; 4 Department of Electronic Science, University of Calcutta, 92, A.P.C. Road, Kolkata 700009, India; 5 Department of Applied Electronics and Instrumentation, Sikkim Manipal Institute of Technology, Majitar, Rangpo, East Sikkim-737136, India; 6 National Institute of Technology, Agartala, Tripura-799055,India

**Keywords:** magnetic quantization, photoemission, quantum dot effective mass superlattices, quantum well effective mass superlattices, quantum well wire effective mass superlattices

## Abstract

The photoemission from quantum wires and dots of effective mass superlattices of optoelectronic materials was investigated on the basis of newly formulated electron energy spectra, in the presence of external light waves, which controls the transport properties of ultra-small electronic devices under intense radiation. The effect of magnetic quantization on the photoemission from the aforementioned superlattices, together with quantum well superlattices under magnetic quantization, has also been investigated in this regard. It appears, taking HgTe/Hg_1−_*_x_*Cd*_x_*Te and In*_x_*Ga_1−_*_x_*As/InP effective mass superlattices, that the photoemission from these quantized structures is enhanced with increasing photon energy in quantized steps and shows oscillatory dependences with the increasing carrier concentration. In addition, the photoemission decreases with increasing light intensity and wavelength as well as with increasing thickness exhibiting oscillatory spikes. The strong dependence of the photoemission on the light intensity reflects the direct signature of light waves on the carrier energy spectra. The content of this paper finds six different applications in the fields of low dimensional systems in general.

## Introduction

With the advent of modern fabrication techniques [1], semiconductors with superlattice structures (SLs) [2], in which alternate layers of two different degenerate materials set up a periodic potential with a periodicity many times the crystal dimensions [3], resulting in energy mini-bands, have been experimentally realized [4]. The SL has been extensively used in many new device structures, such as light emitters [5], electro-optical modulators [6] photo detectors [7], transistors [8], avalanche photodiodes [9], etc. Among the III–V SLs, the GaAs/Ga_1−_*_x_*Al*_x_*As SL has been extensively investigated, in which the GaAs layers form the quantum wells and Ga_1−_*_x_*Al_1−_*_x_*As layers form the potential barriers. The III–V SLs are being extensively used in the realization of high speed optoelectronic devices [10]. The II–VI [11], IV–VI [12] and HgTe/CdTe [13] SLs have also been experimentally realized. The IV–VI SLs shows new physical properties in comparison with the III–V SL owing to the peculiar band structure of the constituent materials [14]. The II–VI SLs are being used for optoelectronic operation in the blue [14]. HgTe/CdTe SLs also find applications for long wavelength infrared detectors and other electro-optical applications [15]. These features arise from the direct band gap compound CdTe whose conduction electrons obey the three band model of Kane and gap-less material HgTe [16]. In this context, it may be noted that in the effective mass SLs, the subbands of the electrons exist in real space [17].

In recent years, the different optical properties of these SLs have been extensively studied on the basis of the assumption that the band structures of the different materials remain an invariant quantity in the presence of intense light field, although this concept is not fundamentally true. In this paper, the photoemission from quantum wells (QWs), quantum well wires (QWWs) and quantum dots (QDs) of effective mass SLs of optoelectronic materials is investigated in the presence of external light waves, which radically change the carrier energy spectrum in a fundamental way on the basis of newly formulated carrier dispersion laws for such quantized systems.

The photoemission from the SLs is a very important quantity from the viewpoint of photoemission spectroscopy [18]. The classical equation of the photo-current density is [19]


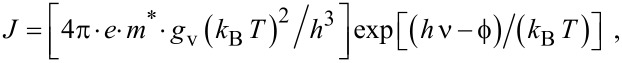


where *e*, m*, *g*_v_, *k*_b_, *T*, *h*, *h*ν, 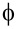 are the electron charge, effective electron mass at the edge of the conduction band, valley degeneracy, the Boltzmann constant, temperature, the Planck constant, incident photon energy along the *z*-axis and work function respectively. It may be noted that the said equation is valid for both charge carriers and in this conventional form the photoemission changes with temperature, work function and the incident photon energy. This relation holds only under the condition of carrier non-degeneracy [20].

The following section gives the theoretical background for this manuscript. In subsection 1, the photoemission from quantum well effective mass SLs of optoelectronic materials under magnetic quantization has been studied on the basis of newly formulated electron dispersion laws. In subsections 2 and 3, the photoemissions from QWW and QD SLs have been investigated. The magneto-photoemission has been studied in subsection 4. The subsection 5 includes six different applications of this paper in the field of superlattices and microstructures in general.

## Theoretical Background

### 1 The formation of photoemission from effective mass quantum well superlattices of optoelectronic materials under magnetic quantization on the basis of a newly formulated electron energy spectrum in the presence of external photo excitation.

The simplified electron energy spectra in optoelectronic materials up to the second order in the presence of intense external light waves, whose unperturbed dispersion relations of the conduction electrons are defined by the three and two band models of Kane together with parabolic energy bands, can be expressed as [21]

[1]
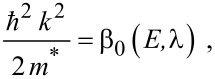


[2]
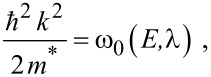


[3]
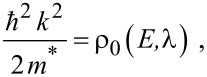


where 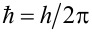, m* is the effective electronic mass at the edge of the unperturbed conduction band, *k* = (*k_x_*, *k_y_*, *k_z_*) is the electronic wave vector,


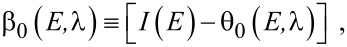



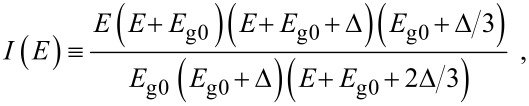


*E* is the electron energy as measured from the edge of the conduction band in the vertically upward direction in the absence of any field, *E*_g0_ is the band gap in the absence of any field, Δ is the spin–orbit splitting constant in the absence of any field,


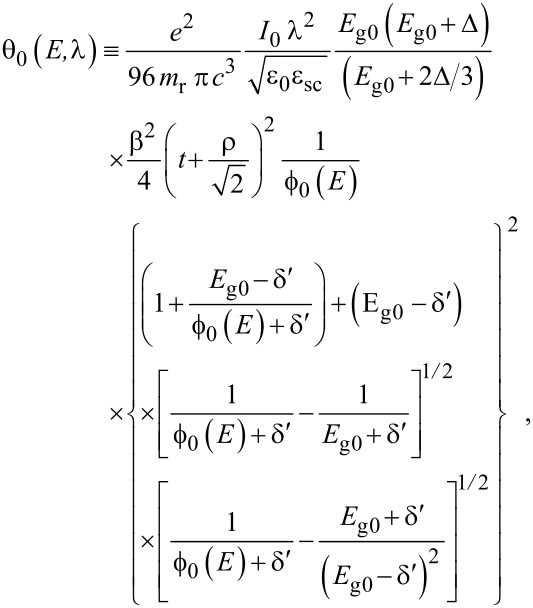


*e* is the magnitude of electronic charge, *m*_r_ is the reduced mass and is given by *m*_r_^−1^ = (m*)^−1^ + *m*_v_^−1^, *m*_v_ is the effective mass of the heavy hole at the top of the valance band in the absence of any field, *I*_0_ is the light intensity of wavelength λ, ε_0_ is the permittivity of vacuum, ε_sc_ is the permittivity of the material,


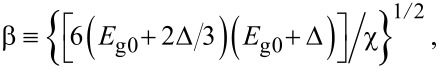



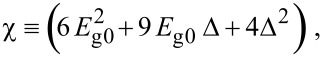



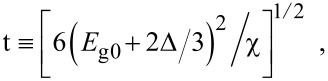



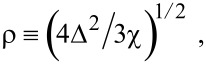



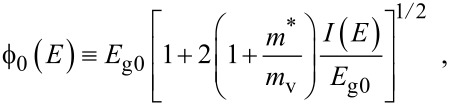



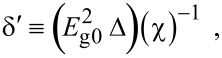



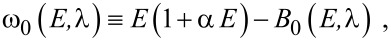



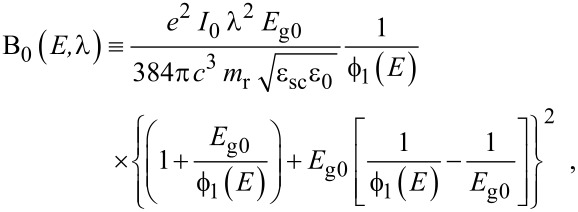



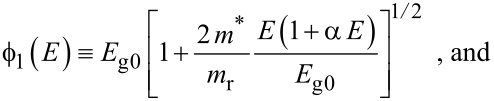



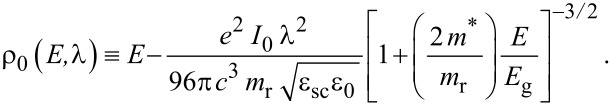


The electron energy spectrum in effective mass superlattices in the presence of a strong external electromagnetic field can be expressed as [17]

[4]
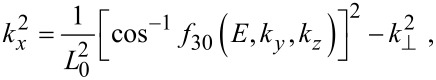


where *L*_0_ ≡ *a*_0_ + *b*_0_ is the SL period, *a*_0_ and *b*_0_ and are the widths of the barrier and the well respectively,


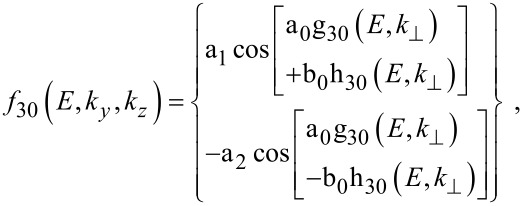



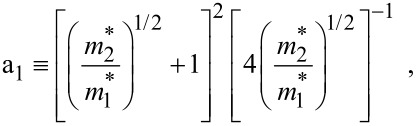



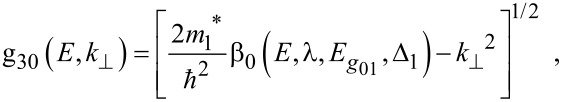



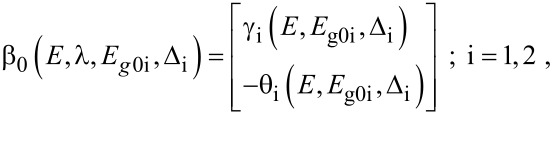



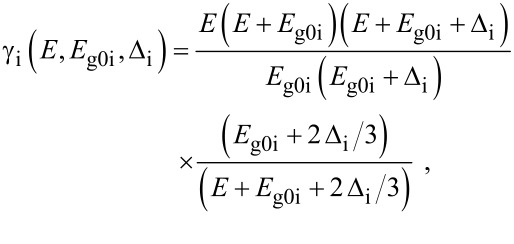



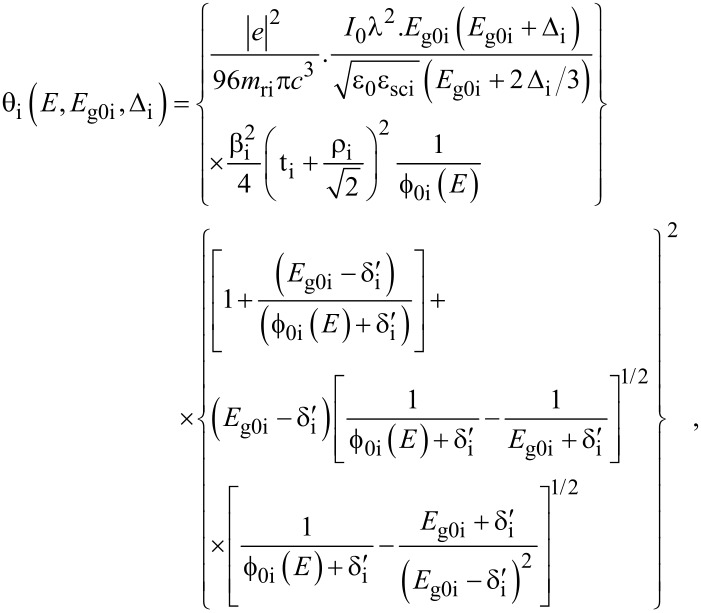



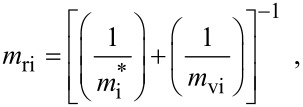



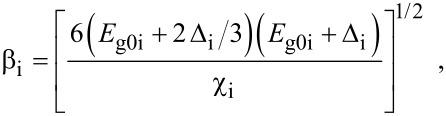



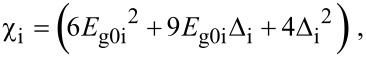



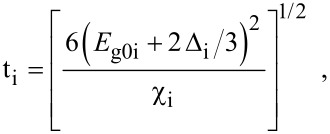



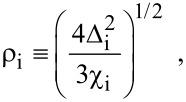



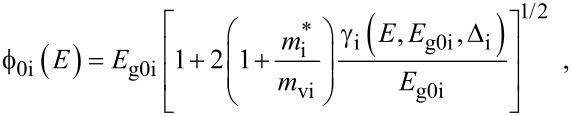



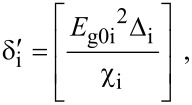



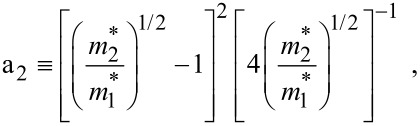



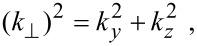



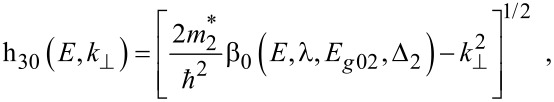


the subscript 1 in the energy band parameters refers to the first material and the subscript 2 refers to the second material of the SL.

When the unperturbed bulk dispersion law of the constituent materials is defined by the two band model of Kane, Equation 4 remains valid and β_0_(*E*, λ, *E*_g0i_, Δ_i_) is replaced by τ_0_(*E*, λ, *E*_g0i_), where


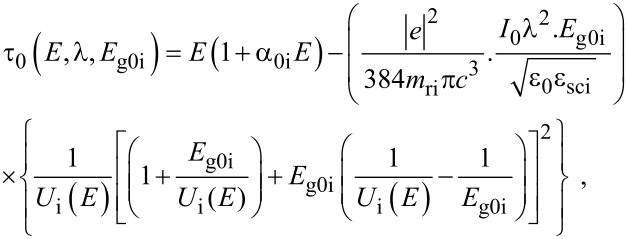



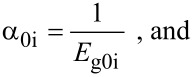



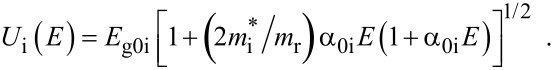


When the unperturbed bulk dispersion law of the constituent materials is defined by parabolic energy bands, Equation 4 remains as it is, and only β_0_(*E*, λ, *E*_g0i_, Δ_i_) is replaced by ρ_0_(*E*, λ, *E*_g0i_), where


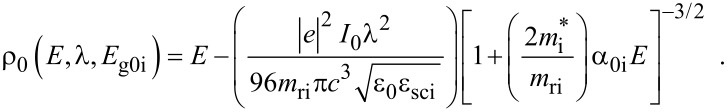


In the presence of a quantizing magnetic field *B* along the *x* direction, the electron dispersion relation in quantum well effective mass superlattices in the present case, is given by

[5]
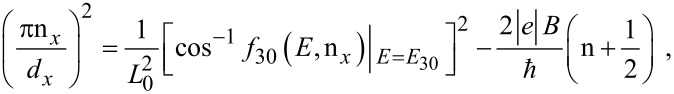


where n*_x_* is the size quantum number along the *x* direction (n*_x_* = 1, 2, 3…), *d_x_* is the nanothickness along the *x* direction, *E*_30_ is the totally quantized energy, n is the Landau quantum number (n = 0, 1, 2, 3…),


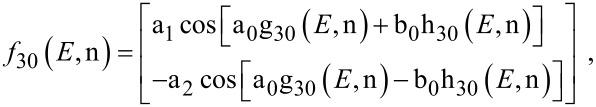



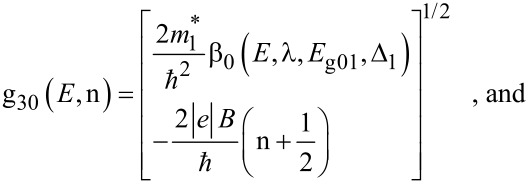



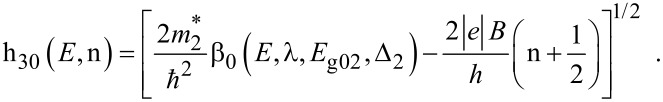


The electron concentration is given by

[6]
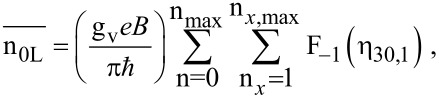



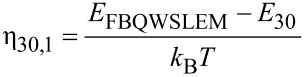


and *E*_FBQWSLEM_ is the Fermi energy in this case and F_−1_(η_30,1_) is the Fermi–Dirac integral of order −1 and is the special case of the Fermi–Dirac integral of order *j* as defined in [22].

The photoelectric current density is given by

[7]
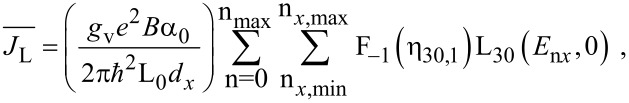


where, α_0_ is the probability of photoemission,


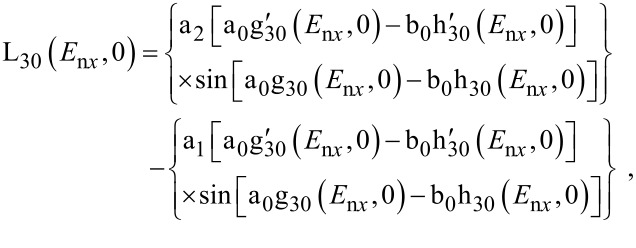



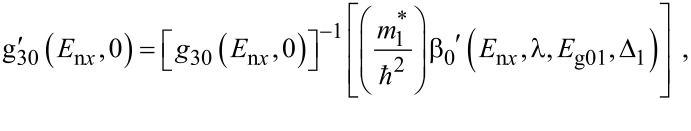



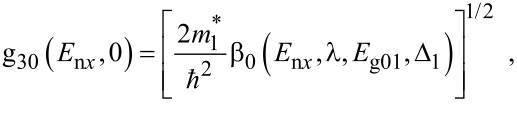



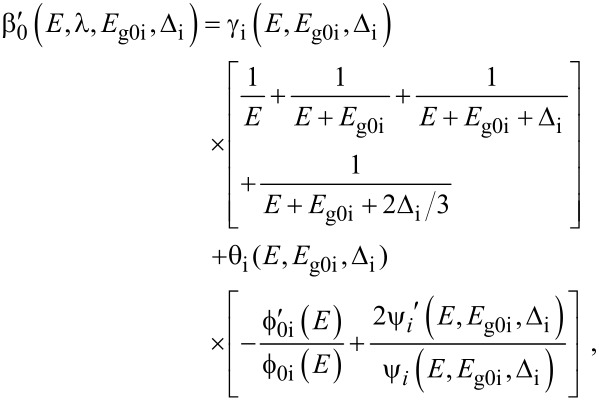



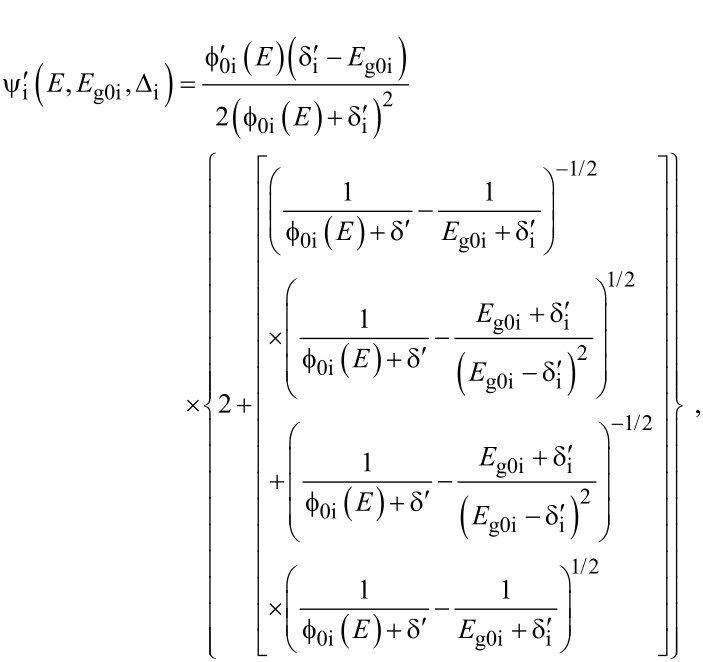



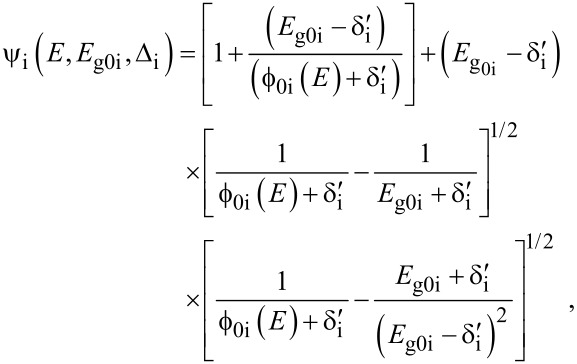



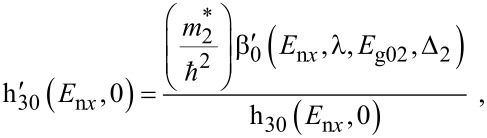



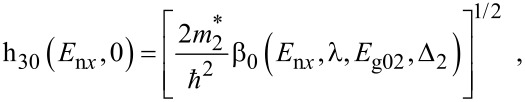



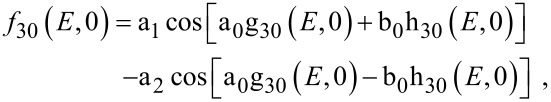



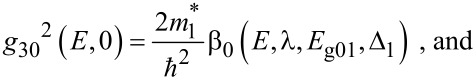



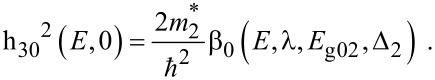


E_n_*_x_* should be determined from the equation

[8]
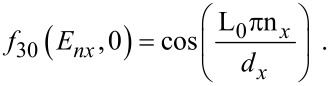


The n*_x_*_,min_ in Equation 7 must be determined from the inequality

[9]
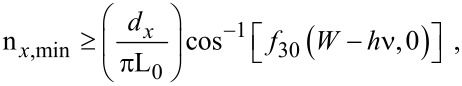


where *W* is the electron affinity.

When the unperturbed bulk dispersion relation of the constituent materials are defined by the two band model of Kane, all the pertinent equations above remain unchanged, where β_0_(*E*, λ, *E*_g0i_, Δ_i_) is to be replaced by τ_0_(*E*, λ, *E*_g0i_) and β'_0_ (*E*, λ, *E*_g0i_, Δ_i_) should be replaced by τ'_0_ (*E*, λ, *E*_g0i_) where


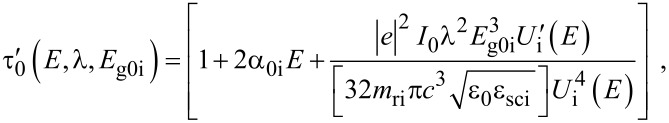


with


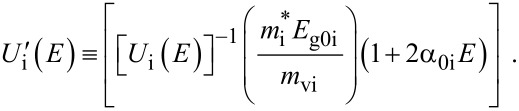


For the perturbed parabolic bulk dispersion relation of the constituent materials in this case β_0_(*E*, λ, *E*_g0i_, Δ_i_) should be replaced by ρ_0_(*E*, λ, *E*_g0i_) and β'_0_ (*E*, λ, *E*_g0i_, Δ_i_) should be replaced by ρ'_0_ (*E*, λ, *E*_g0i_), where


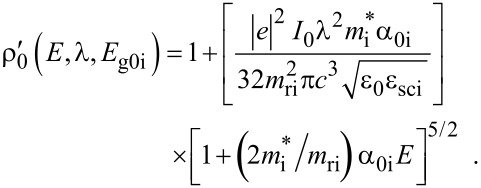


### 2 Photoemission from quantum well wire effective mass superlattices

The electron dispersion relation in this case is given by

[10]
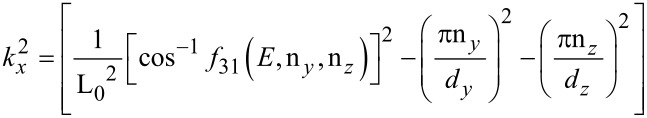


where, n*_y_* and n*_z_* are size quantum numbers along the *y* and *z* directions, *d_y_* and *d_z_* are the nano-thicknesses along the *y* and *z* directions,


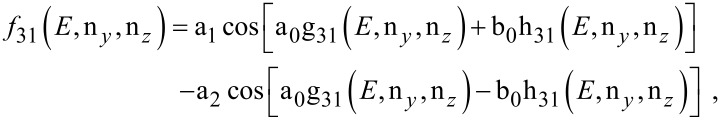



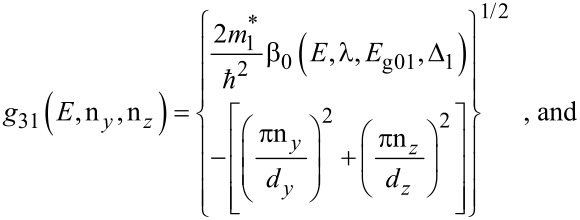



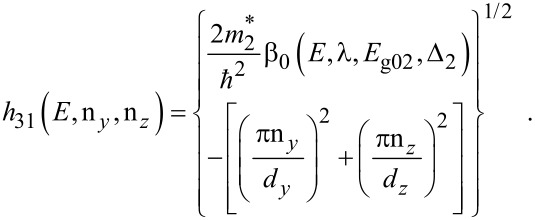


The sub-band energy *E*_31_ can be written as

[11]
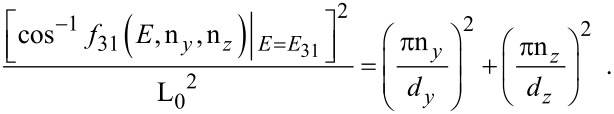


The photo-emitted current assumes the form

[12]
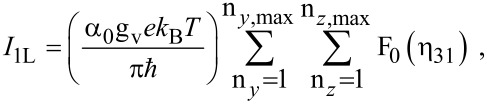


where


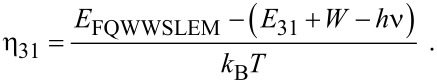


The electron concentration per unit length is given by

[13]
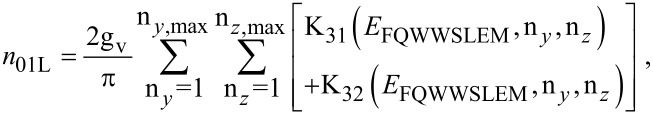



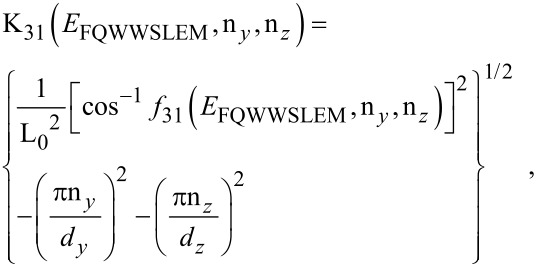



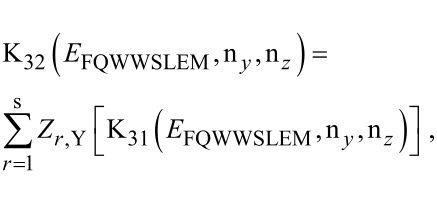



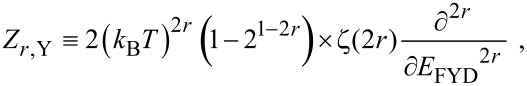


ζ(2*r*) is the zeta function of order 2*r*, s is the upper limit of the summation and Y = QWWSLEM. For the cases defined by the perturbed two band model of Kane or a parabolic energy band, β_0_(*E*, λ, *E*_g0i_, Δ_i_) should be replaced by τ_0_(*E*, λ, *E*_g0i_) and ρ_0_(*E*, λ, *E*_g0i_) respectively. The basic forms of Equation 12 and Equation 13 remain unchanged.

### 3 Photoemission from quantum dot effective mass superlattices

The electron energy spectrum in this case can be expressed as

[14]
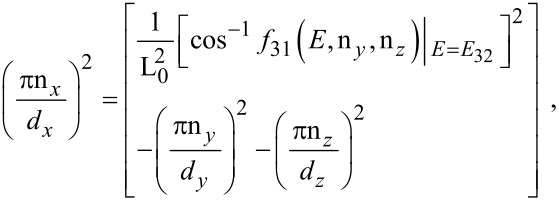


where *E*_32_ is the totally quantized electron energy in this case. The electron concentration can be written as

[15]
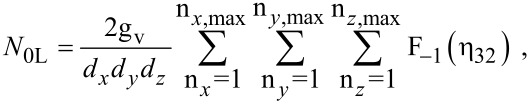


where η_32_ = (*E*_FQDSLEM_ – *E*_32_)/*k*_B_*T*, in which *E*_FQDSLEM_ is the Fermi energy in the present case.

The photo-emitted current density assumes the form

[16]
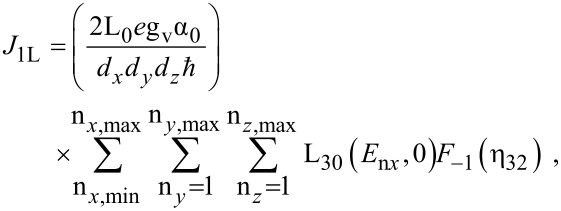


where


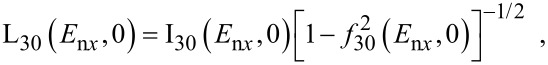


I_30_(*E*_n_*_x_*,0) and *f*_30_(*E*_n_*_x_*,0) are defined in connection with Equation 7 and

[17]
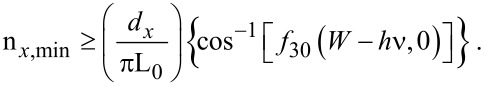


Besides, the *E*_n_*_x_* should be determined from the equation

[18]
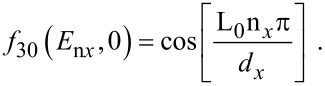


### 4 Magneto-photoemission from effective mass superlattices

The electron dispersion law in this case assumes the form

[19]
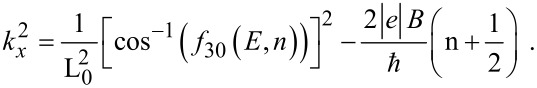


The Landau level energy *E*_33_ is given by

[20]
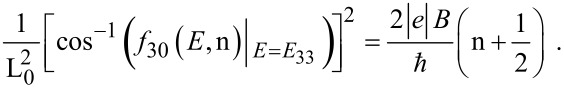


The photo-emitted current density can be written as

[21]
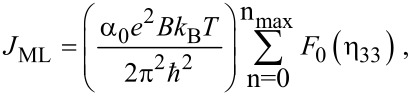


where η_33_ = [*E*_FBSLEM_ –(*E*_33_ + W − hν)]/*k*_B_*T*, in which *E*_FBSLEM_ is the Fermi energy in the present case.

The electron concentration can be expressed as

[22]
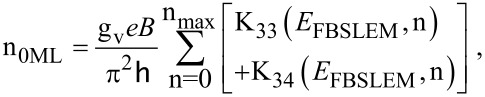



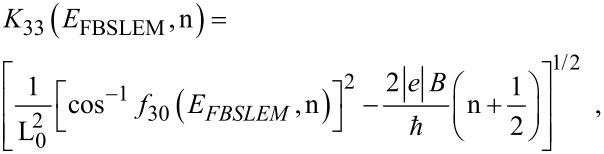



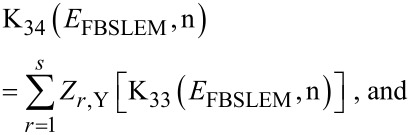


Y = BSLEM.

### 5 The six different applications of the results of this paper in the field of nanostructured electronics in general

The investigations as presented in this paper find six different applications in the realm of modern quantum effect devices and they are briefly written as follow:

#### 5.1 Debye screening length

The Debye screening length (DSL) of the carriers in the semiconductors is a fundamental quantity, characterizing the screening of the Coulomb field of the ionized impurity centers by the free carriers. It affects many special features of the modern semiconductor devices, the carrier mobility under different mechanisms of scattering, and the carrier plasmas in semiconductors [23]. The DSL (*L*_D_) can, in general, be written as [24]

[23]
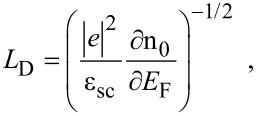


where n_0_ and *E*_F_ are applicable for bulk samples.

The thermoelectric power of the carriers in semiconductors in the presence of a classically large magnetic field is independent of scattering mechanisms and is determined only by their energy band spectra [25]. The magnitude of the thermoelectric power *G* can be written under the condition of carrier degeneracy [25] as

[24]
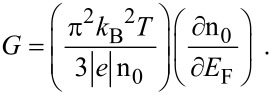


Using Equation 23 and Equation 24, one obtains

[25]
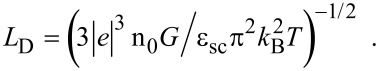


Therefore, we can experimentally determine *L*_D_ by knowing the experimental curve of *G* versus carrier concentration at a fixed temperature. It is evident that the DSL for a system can be investigated for different cases by using the functional dependence between the electron concentration and the Fermi energy, as given for different cases in the different sections of this paper.

#### 5.2 Carrier contribution to the elastic constants

The knowledge of the carrier contribution to the elastic constants is very important in studying the mechanical properties of the materials and has been investigated in the literature [26]. The electronic contribution to the second- and third- order elastic constants can be written as [27]

[26]
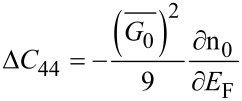


and

[27]
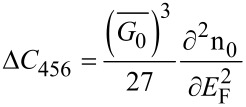


where 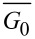 is the deformation potential constant. Thus, using Equation 26, Equation 27 and Equation 24, we can write

[28]
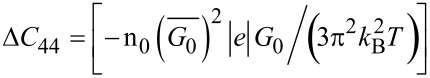


and

[29]
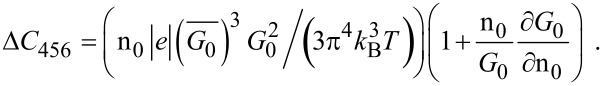


Thus, again the experimental graph of *G*_0_ versus n_0_ allows us to determine the electronic contribution to the elastic constants for materials having arbitrary spectra.

#### 5.3 Effective electron mass

The concept of effective mass of the carriers in different materials, being connected with the mobility, is known to be one of the most important pillars in the whole field of solid state and related sciences and is used for the analysis of the semiconductor devices under different operating conditions in general [28]. It must be noted that among the various definitions of the effective electron mass [29], it is the effective momentum mass that should be regarded as the basic quantity [30]. This is due to the fact that it is this mass which appears in the description of transport phenomena and all other properties of the conduction electrons of the semiconductors having arbitrary dispersion laws [31]. It is the effective momentum mass which enters in various transport coefficients and plays the most dominant role in explaining the experimental results under different scattering mechanisms [32]. The carrier degeneracy in semiconductors influences the effective mass when it is energy dependent. Under degenerate conditions, only the electrons at the Fermi surface of n-type semiconductors participate in the conduction process and hence, the effective momentum mass of the electrons (EMM), corresponding to the Fermi level, would be of interest in electron transport under such conditions. The Fermi energy is again determined by the carrier energy spectrum and the carrier concentration and therefore, these two features would determine the dependence of the EMM in degenerate materials on the degree of carrier degeneracy. In recent years, the EMM in such materials under different external conditions has been studied extensively [33]. It has different values in different materials and varies with electron concentration, with the magnitude of the reciprocal quantizing magnetic field under magnetic quantization, with the quantizing electric field as in inversion layers, with the nano-thickness as in quantum wells and quantum well wires and with superlattice period as in the quantum confined superlattices having various carrier energy spectra.

The expression of the EMM in the *i*-th direction is given by

[30]
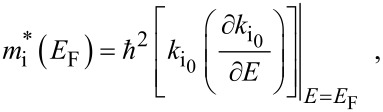


where i_0_ = *x*, *y* and *z*.

From the different sections of this paper, the EMM can be formulated by using the respective dispersion relation, and their dependencies with respect to various variables can also be studied. In addition to Fermi energy and other system constraints, the effective mass will depend on the respective quantum numbers, which is the characteristic feature of effective mass superlattices.

#### 5.4 Diffusivity to mobility ratio

The diffusivity (*D*) to mobility (μ) ratio (DMR) of the carriers in semiconductor devices is known to be very useful [34] since the diffusion constant (a quantity often used in device analysis but whose exact experimental determination is rather difficult) can be obtained from this ratio by knowing the experimental values of the mobility. In addition, it is more accurate than any of the individual relations for the diffusivity or the mobility, which are the two most widely used quantities for the characterization of carrier transport in modern nanostructured materials and devices. The classical DMR equation is valid for both types of carriers. In its conventional form, the DMR increases linearly with the temperature *T*_,_ being independent of the carrier concentration. This relation holds only under the condition of carrier non-degeneracy although its validity has been suggested erroneously for degenerate materials [35]. The performance of the electron devices at the device terminals and the speed of operation of modern switching transistors are significantly influence by the degree of carrier degeneracy present in these devices [36]. The simplest way of analyzing them under degenerate conditions is to use the appropriate DMR to express the performance of the devices at the device terminals and the switching speed in terms of the carrier concentration [22].

It is well known from the fundamental work of Landsberg [37] that the DMR for electronic materials having degenerate electron concentration is essentially determined by their respective energy band structures. It can, in general, be proved that for bulk specimens the DMR is given by [38]

[31]
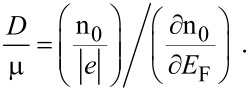


Under the electric quantum limit, as in inversion layers and *n*-*i*-*p*-*i* structures, referring to the lowest electric sub-band, Equation 31 assumes the form [22]

[32]
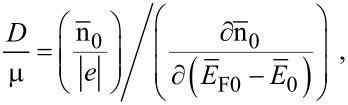


where 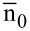 and 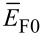 and 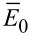 are the electron concentration, the energy of the electric sub-band and the Fermi energy in the electric quantum limit.

For inversion layers and *n-i-p-i* structures, under the condition of electric quantum limit, Equation 24 can be expressed as [25]

[33]
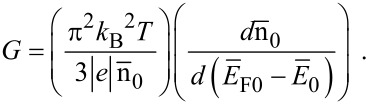


Using the appropriate equations one obtains

[34]
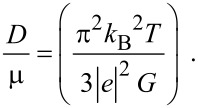


Thus, the DMR for degenerate materials can be determined by knowing the experimental values of *G*. The suggestion for the experimental determination of the DMR for degenerate semiconductors having arbitrary dispersion laws, as given by Equation 34, does not contain any energy band constants. For a fixed temperature, the DMR varies inversely as *G*. Only the experimental values of *G*, for any material as a function of electron concentration, will generate the experimental values of the DMR for that range of n_0_ for that system. Since *G* decreases with increasing n_0_, from Equation 34 one can infer that the DMR will increase with increasing n_0_. This statement is the compatibility test so far as the suggestion for the experimental determination of DMR for degenerate materials is concerned.

#### 5.5 Third order nonlinear optical susceptibility

The third order nonlinear optical susceptibility can be written as [38]

[35]
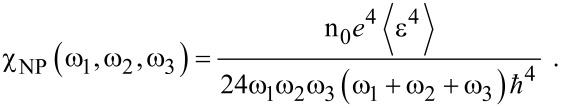


where


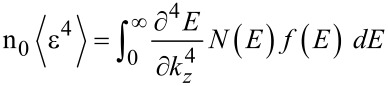


and the other notations are defined in [38]. The term 
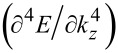
 can be formulated by using the dispersion relations of different materials as given in appropriate sections of this paper. Thus one can investigate the χ_NP_(ω_1_, ω_2_, ω_3_) for all materials as considered in this paper.

#### 5.6 Generalized Raman gain

The generalized Raman gain in optoelectronic materials can be expressed as [39]

[36]
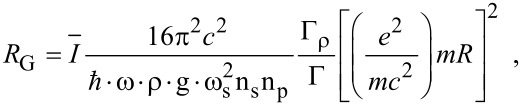


where


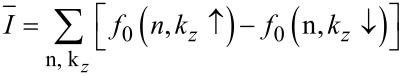



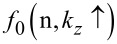
 is the Fermi factor for spin-up Landau levels, 
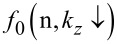
 is the Fermi factor for spin down Landau levels and the other notations are defined in [39]. It appears then that the formulation of *R*_G_ is determined by the appropriate derivation of *I*, which in turn requires the magneto-dispersion relations. By using the appropriate formulas *R*_G_ can, in general, be investigated.

Thus we can summarize the whole theoretical background in the following way. In this paper, we have studied the photoemission from quantum wire and quantum dot effective mass superlattices of optoelectronic materials, on the basis of newly formulated electron dispersion relations, in the presence of external photo-excitation. In addition, the influence of magnetic field on the photoemission from the said superlattices, together with quantum well effective mass super lattices in the presence of quantizing magnetic field, has also been studied in this context. The strong dependences of the photoemission on the light intensity reflect the direct signature of light waves on the carrier energy spectra. The content of this paper finds six real applications in the field of nanoscience and technology in general.

## Results and Discussion

**Figure 1:**
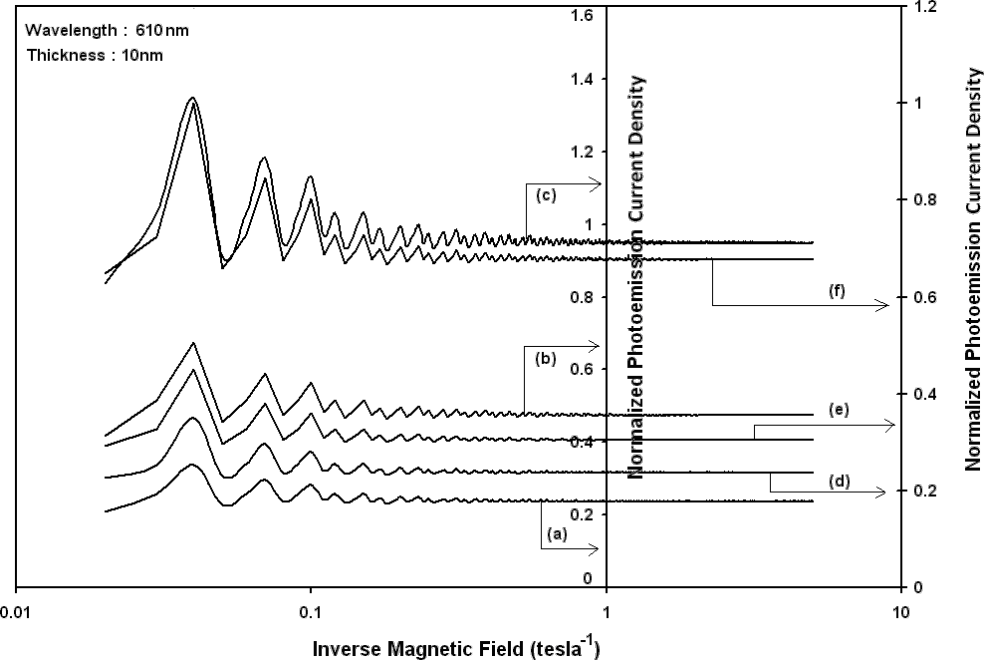
Plot of the normalized photoemission current density from QW effective mass superlattices of HgTe/Hg_1−_*_x_*Cd*_x_*Te as a function of inverse magnetic field, in which the curves (a), (b) and (c) represent the perturbed three and two band models of Kane and parabolic energy bands, respectively. The curves (d), (e) and (f) exhibit the corresponding plots of In*_x_*Ga_1−_*_x_*As/InP.

**Figure 2:**
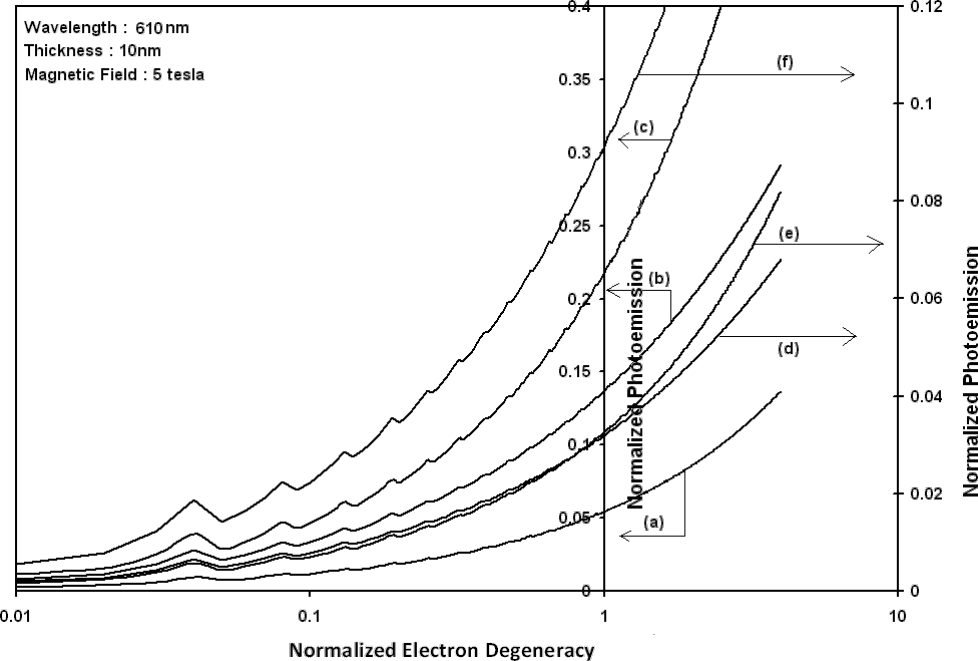
Plot of the normalized photoemission as a function of normalized electron degeneracy for all cases of Figure 1.

Using Equation 6 and Equation 7 and taking the numerical values of the energy band constants from [19], the normalized photo-emitted current density from QW HgTe/Hg_1−_*_x_*Cd*_x_*Te effective mass SL, whose constituent materials obey the perturbed three band model of Kane in the presence of external photo-excitation, has been plotted as a function of inverse quantizing magnetic field, as shown in plot (a) of Figure 1. The curves (b) and (c) of the same figure have been drawn for the perturbed two band model of Kane and that of perturbed parabolic energy bands respectively. The curves (d), (e) and (f) in the same figure exhibit the corresponding plots of QW In*_x_*Ga_1−_*_x_*As/InP effective mass SL. Figure 2 to Figure 5 show the variations of the normalized photo-emitted current density from the said SLs as a function of normalized electron degeneracy, normalized intensity, wavelength and thickness, respectively, for all the cases of Figure 1. Using Equation 15 and Equation 16, the normalized photocurrent from QWW effective mass HgTe/Hg_1−_*_x_*Cd*_x_*Te SL, whose constituent materials obey the perturbed three band model of Kane in the presence of external light waves, has been depicted in plot (a) of Figure 6 as a function of film thickness. The curves (b) and (c) of the same figure have been drawn for the perturbed two band model of Kane and perturbed parabolic energy bands respectively. The curves (d), (e) and (f) in the same figure exhibit the corresponding plots of In*_x_*Ga_1−_*_x_*As/InP QWW effective mass SL.

**Figure 3:**
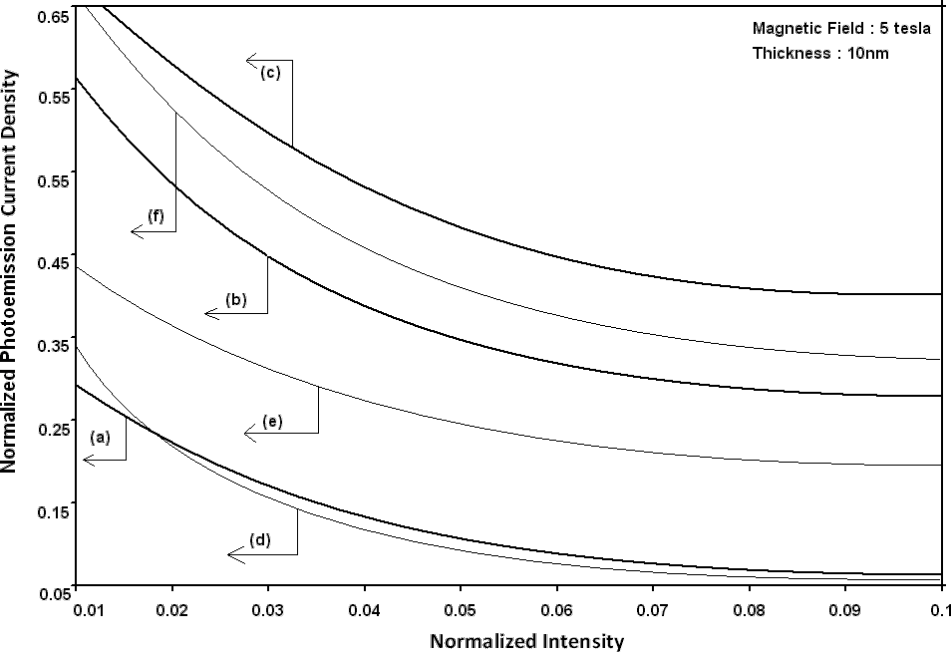
Plot of the normalized photoemission current density as a function of normalized light intensity for all cases of Figure 1.

**Figure 4:**
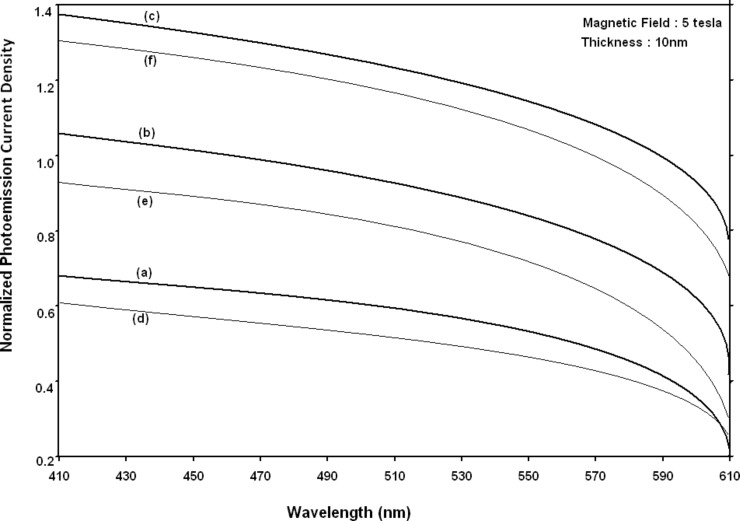
Plot of the normalized photoemission current density as a function of wavelength for all cases of Figure 1.

**Figure 5:**
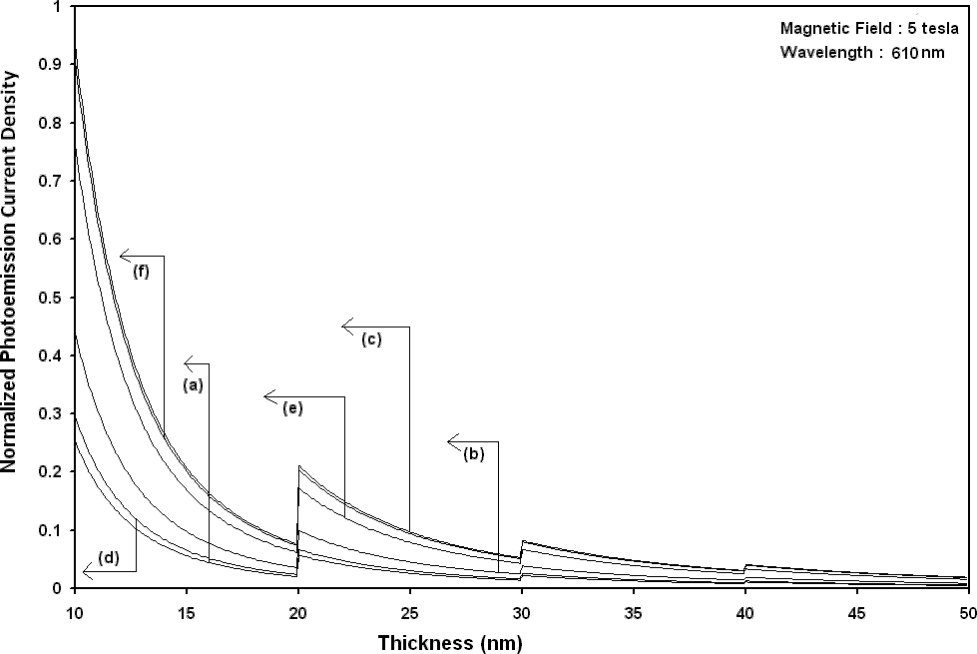
Plot of the normalized photoemission current density as a function of film thickness for all cases of Figure 1.

**Figure 6:**
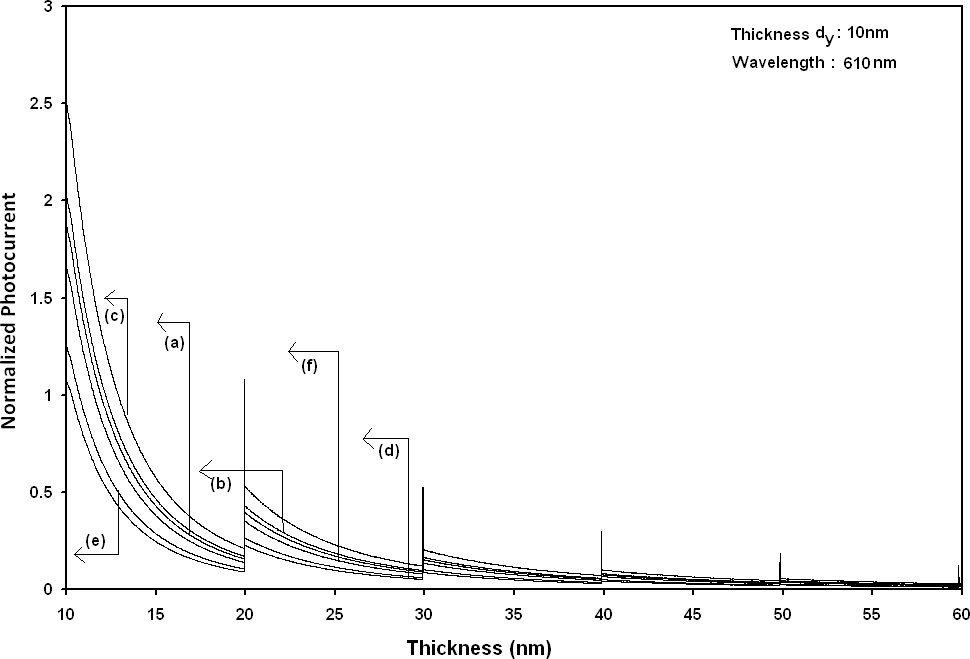
Plot of the normalized photocurrent from quantum well wire effective mass superlattices of HgTe/Hg_1−_*_x_*Cd*_x_*Te as a function of film thickness in which the curves (a), (b) and (c) represent the perturbed three and two band models of Kane and parabolic energy bands, respectively. The curves (d), (e) and (f) exhibit the corresponding plots of In*_x_*Ga_1−_*_x_*As/InP.

**Figure 7:**
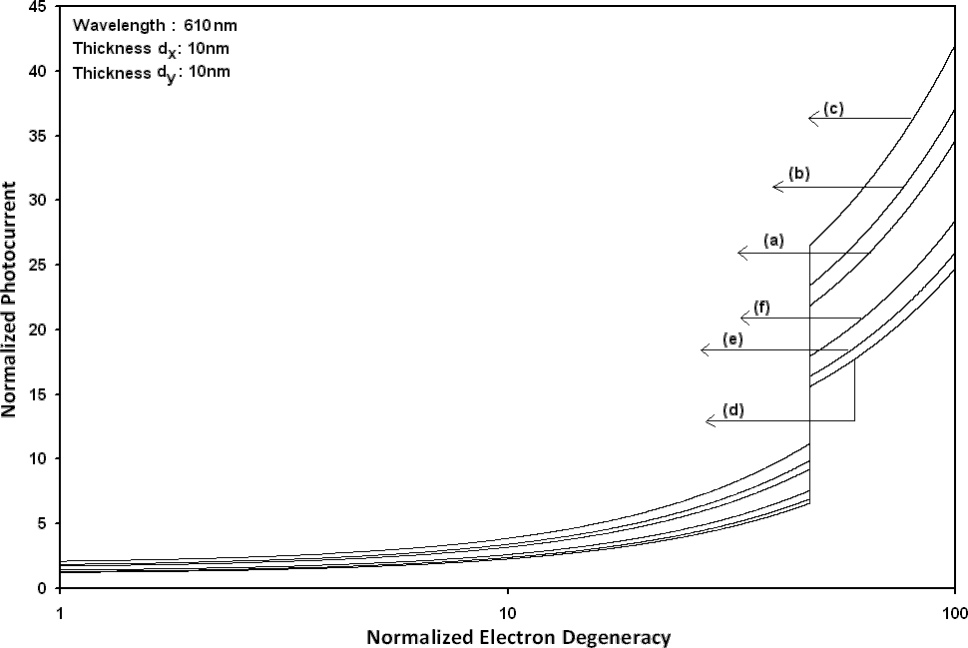
Plot of the normalized photocurrent from quantum well wire effective mass superlattices of HgTe/Hg_1−_*_x_*Cd*_x_*Te and In*_x_*Ga_1−_*_x_*As/InP as a function of normalized electron degeneracy for all cases of Figure 6.

Figure 7 to Figure 10 exhibit the plots of the normalized photo-emitted current as functions of normalized electron degeneracy, normalized intensity, wavelength and normalized incident photon energy, respectively, for all the cases of Figure 6.

**Figure 8:**
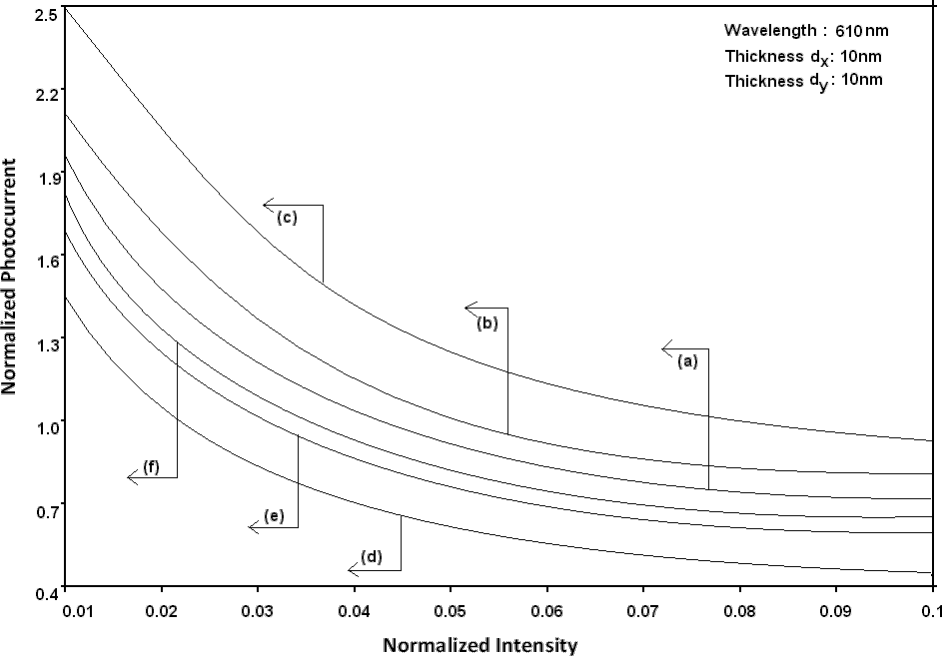
Plot of the normalized photocurrent from quantum well wire effective mass superlattices of HgTe/Hg_1−_*_x_*Cd*_x_*Te and In*_x_*Ga_1−_*_x_*As/InP as a function of normalized intensity for all cases of Figure 6.

**Figure 9:**
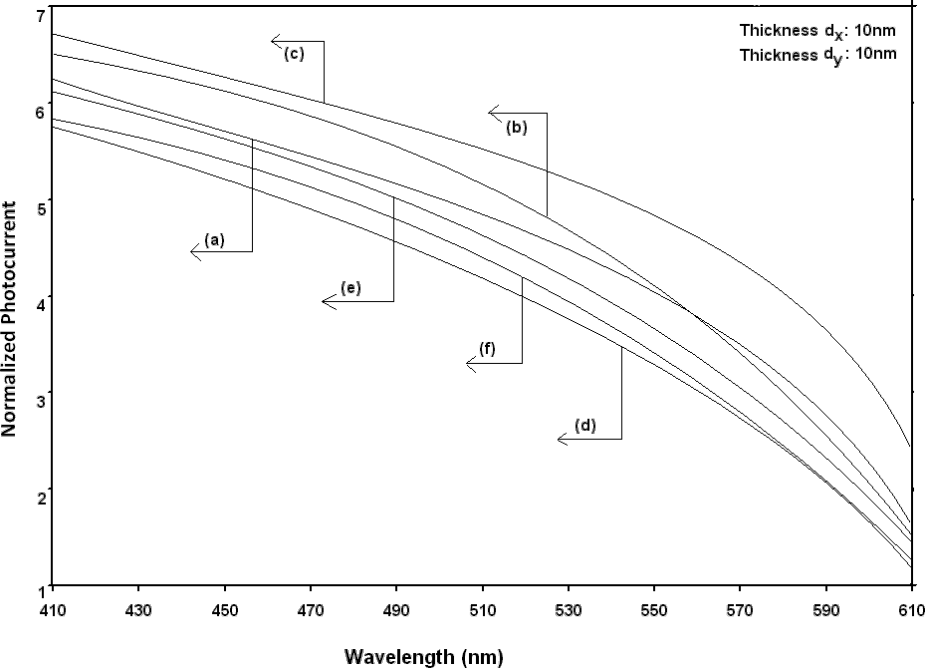
Plot of the normalized photocurrent from quantum well wire effective mass superlattices of HgTe/Hg_1−_*_x_*Cd*_x_*Te and In*_x_*Ga_1−_*_x_*As/InP as a function of light wavelength for all cases of Figure 6.

**Figure 10:**
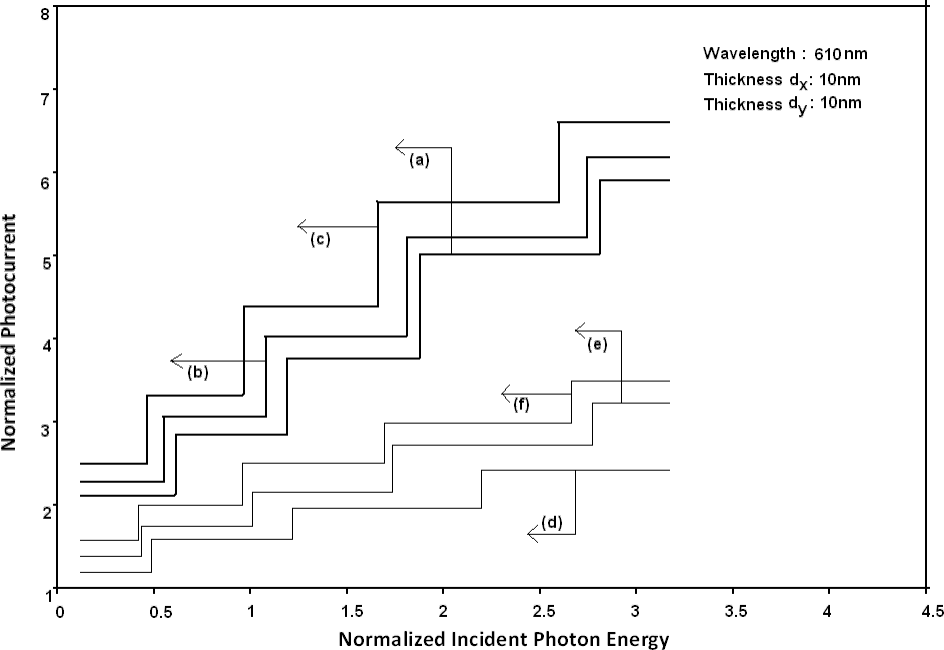
Plot of the normalized photocurrent as a function of normalized incident photon energy from quantum well wire effective mass superlattices of HgTe/Hg_1−_*_x_*Cd*_x_*Te and In*_x_*Ga_1−_*_x_*As/InP for all cases of Figure 6.

**Figure 11:**
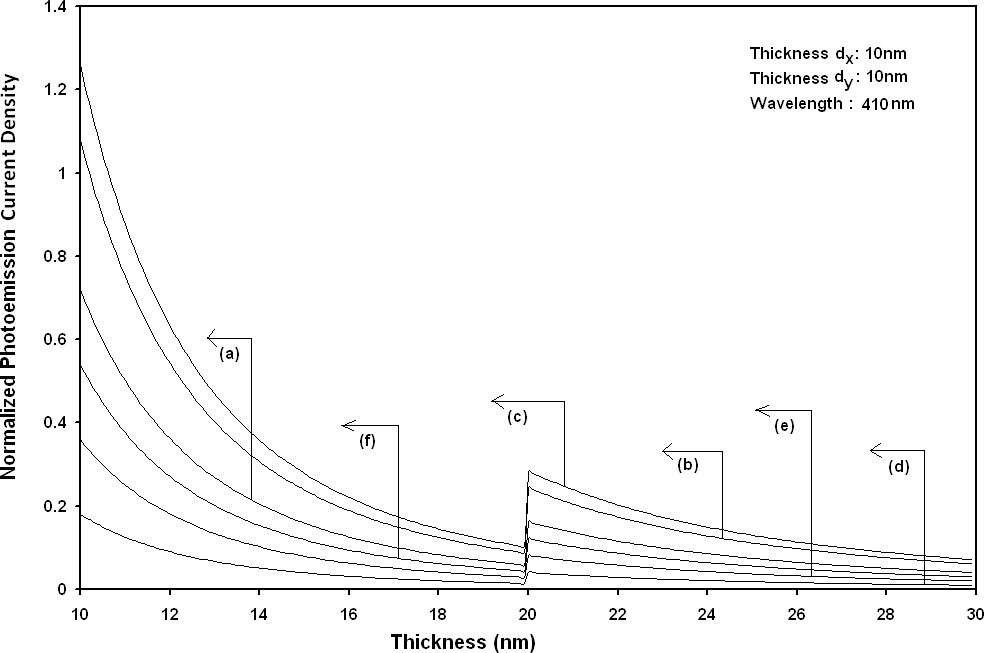
Plot of the normalized photoemission current density from quantum dot effective mass superlattices of HgTe/Hg_1−_*_x_*Cd*_x_*Te as a function of film thickness in which the curves (a), (b) and (c) represent the perturbed three and two band models of Kane and parabolic energy bands, respectively. The curves (d), (e) and (f) exhibit the corresponding plots of In*_x_*Ga_1−_*_x_*As/InP.

**Figure 12:**
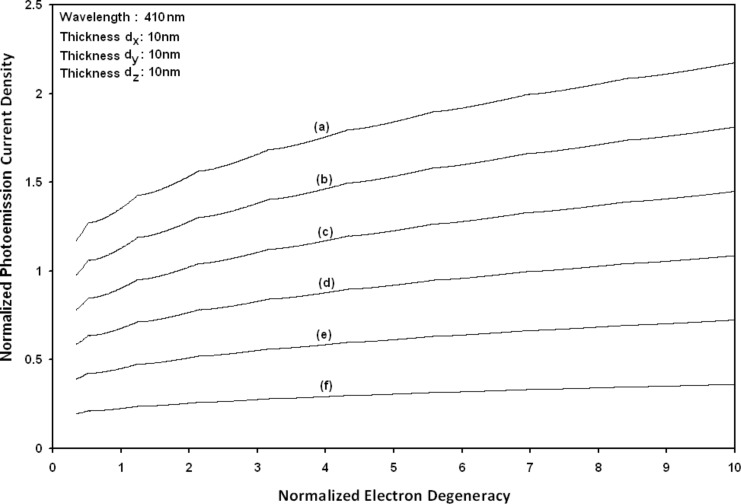
Plot of the normalized photoemission current density from quantum dot effective mass superlattices of HgTe/Hg_1−_*_x_*Cd*_x_*Te and In*_x_*Ga_1−_*_x_*As/InP as a function of normalized electron degeneracy for all cases of Figure 11.

Using Equation 16 and Equation 17, the normalized photo-emitted current density from HgTe/Hg_1−_*_x_*Cd*_x_*Te and In*_x_*Ga_1−_*_x_*As/InP effective mass QD SLs respectively has been plotted for all types of band models as a function of film thickness, as shown in Figure 11. Figure 12 to Figure 15 exhibit the plots of normalized photo-emitted current density from the said SLs as functions of normalized electron degeneracy, normalized intensity, wavelength and normalized incident photon energy, respectively, for all cases of Figure 11.

**Figure 13:**
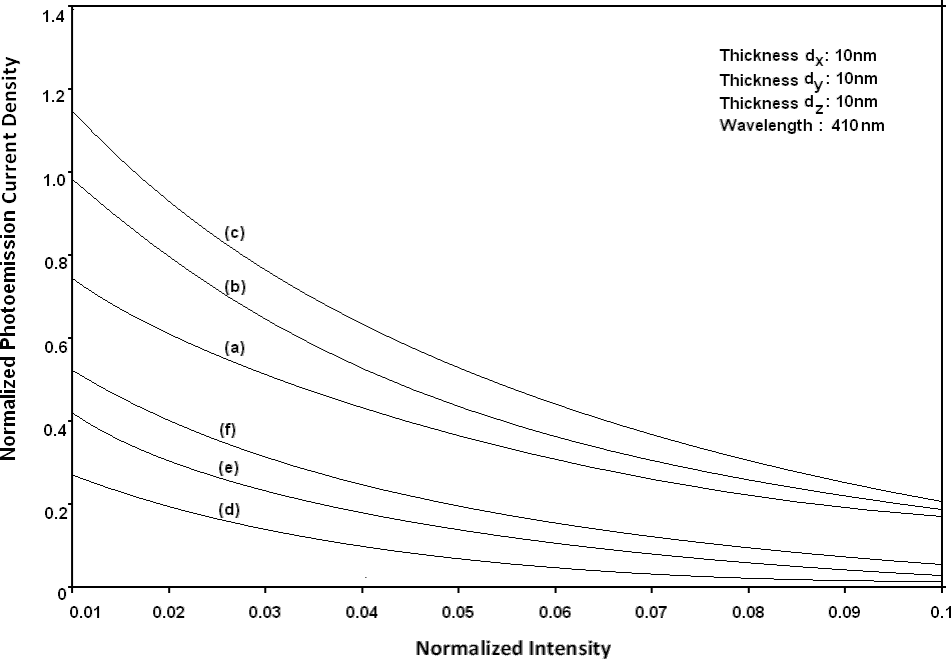
Plot of the normalized photoemission current density from quantum dot effective mass superlattices of HgTe/Hg_1−_*_x_*Cd*_x_*Te and In*_x_*Ga_1−_*_x_*As/InP as a function of normalized light intensity for all cases of Figure 11.

**Figure 14:**
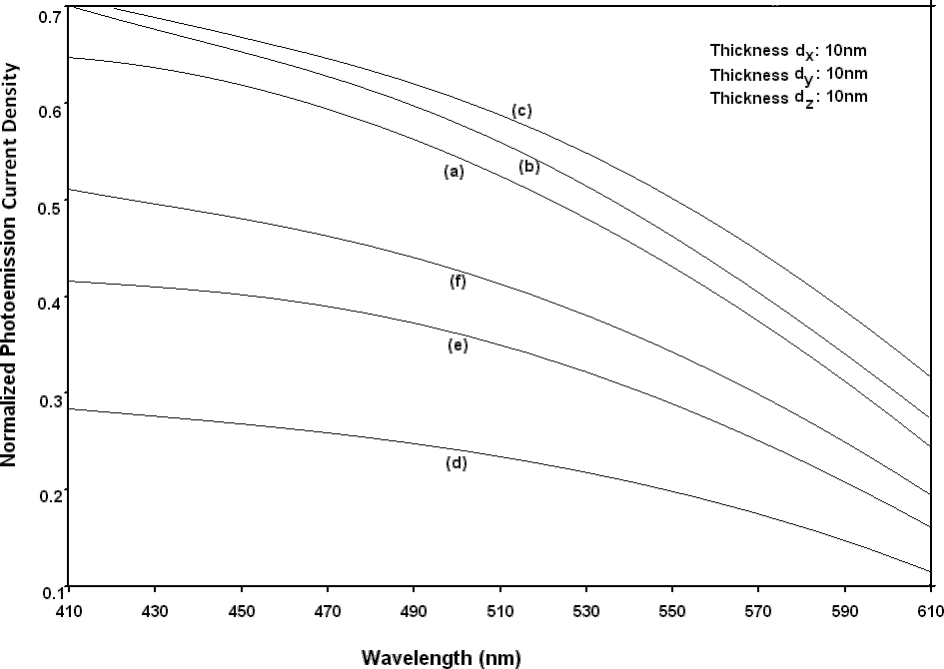
Plot of the normalized photoemission current density from quantum dot effective mass superlattices of HgTe/Hg_1−_*_x_*Cd*_x_*Te and In*_x_*Ga_1−_*_x_*As/InP as a function of light wavelength for all cases of Figure 11.

**Figure 15:**
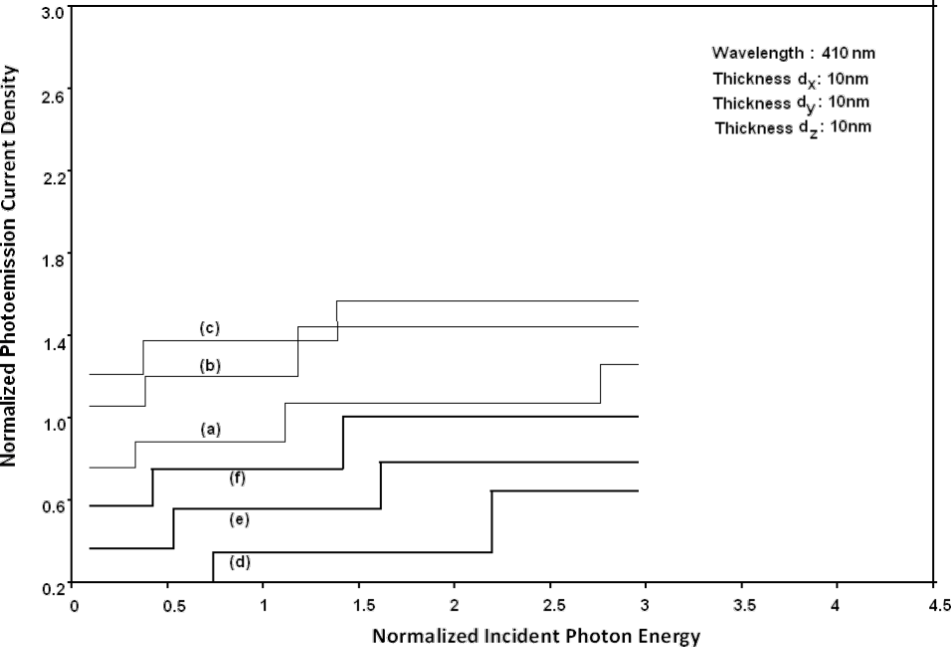
Plot of the normalized photoemission current density from quantum dot effective mass superlattices of HgTe/Hg_1−_*_x_*Cd*_x_*Te and In*_x_*Ga_1−_*_x_*As/InP as a function of normalized incident photon energy for all cases of Figure 11.

**Figure 16:**
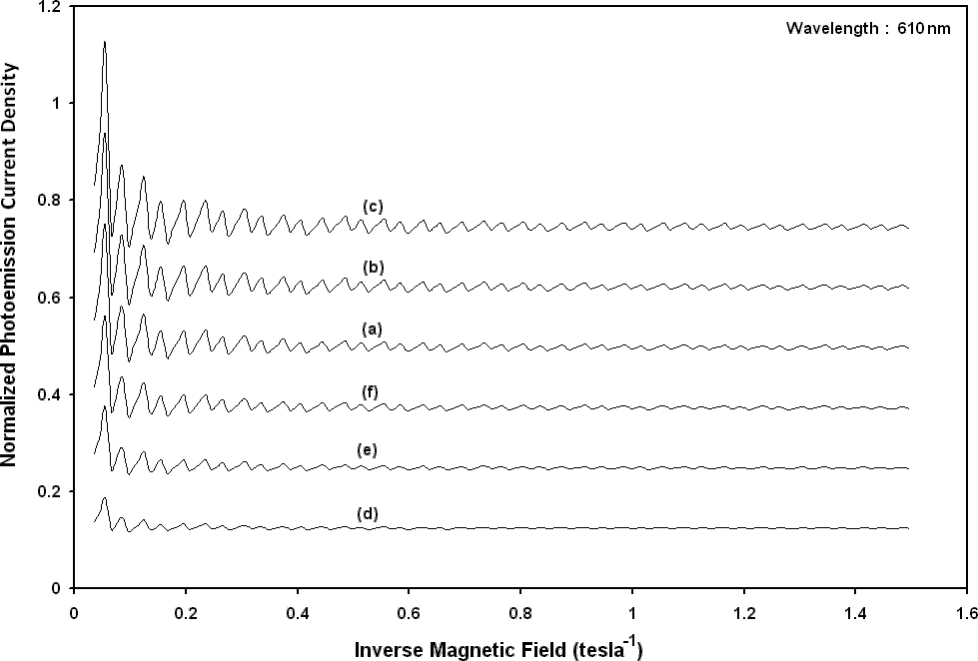
Plot of the normalized photoemission current density from effective mass superlattices of HgTe/Hg_1−_*_x_*Cd*_x_*Te as a function of inverse magnetic field and in which the curves (a), (b) and (c) represent the perturbed three and two band models of Kane and parabolic energy bands, respectively. The curves (d), (e) and (f) exhibit the corresponding plots of In*_x_*Ga_1−_*_x_*As/InP.

Using Equation 21 and Equation 22, the normalized photo-emitted current density from effective mass HgTe/Hg_1−_*_x_*Cd*_x_*Te SL under magnetic quantization, whose constituent materials obey the perturbed three band model of Kane in the presence of external photo-excitation, has been plotted as a function of quantizing inverse magnetic field as shown in plot (a) of Figure 16. The curves (b) and (c) of the same figure have been drawn for perturbed two band model of Kane and perturbed parabolic energy bands, respectively. The curves (d), (e) and (f) in the same figure exhibit the corresponding plots of In*_x_*Ga_1−_*_x_*As/InP SL.

**Figure 17:**
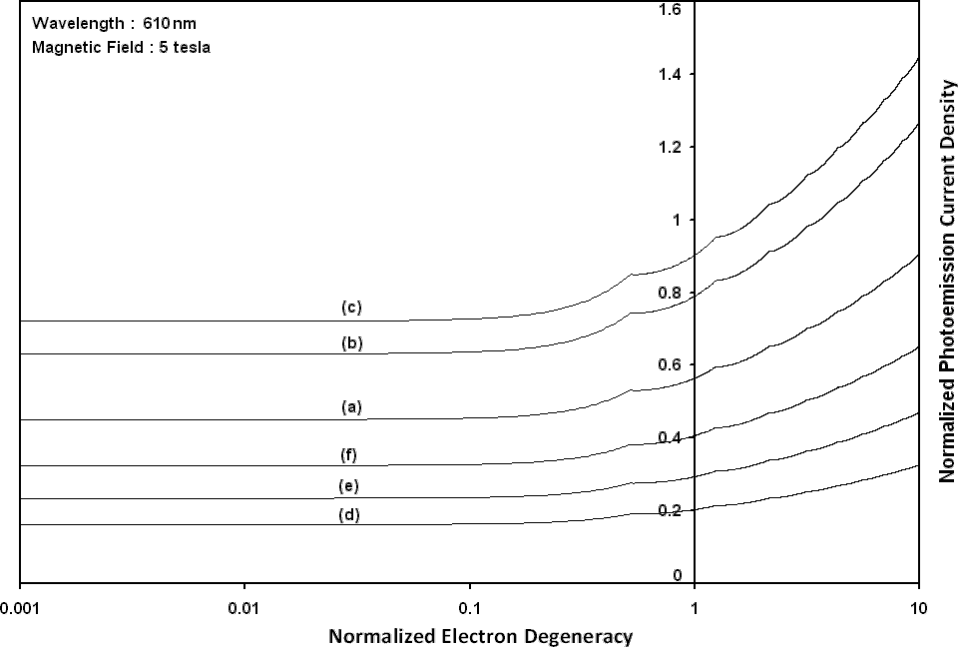
Plot of the normalized magneto photoemission current density from effective mass superlattices of HgTe/Hg_1−_*_x_*Cd*_x_*Te and In*_x_*Ga_1−_*_x_*As/InP as a function of normalized electron degeneracy for all cases of Figure 16.

Figure 17 to Figure 20 exhibit the said variation in this case as functions of normalized electron degeneracy, normalized intensity, wavelength and normalized incident photon energy, respectively, for all the cases of Figure 16.

**Figure 18:**
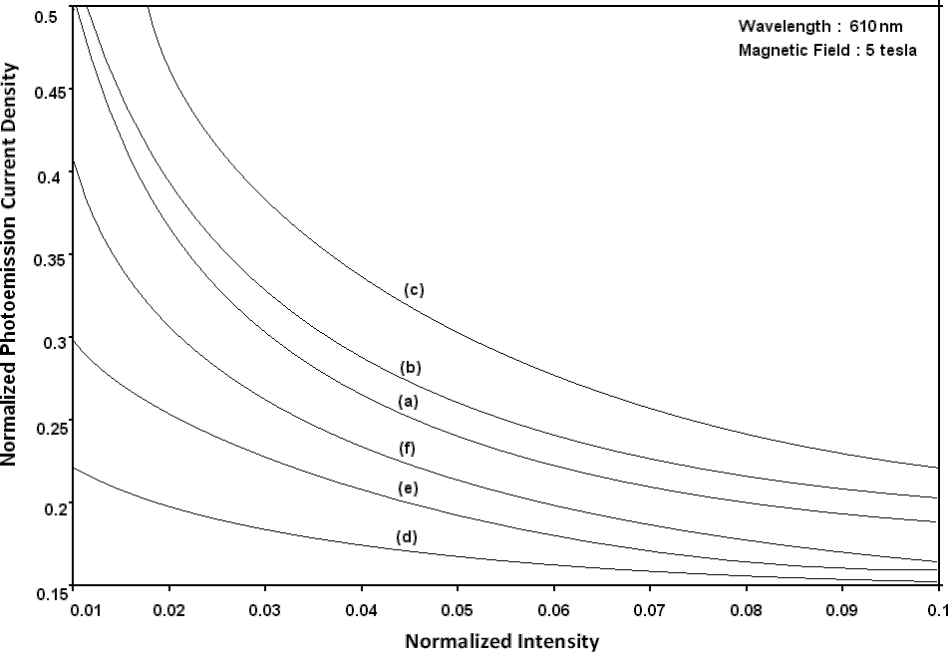
Plot of the normalized magneto photoemission current density from effective mass superlattices of HgTe/Hg_1−_*_x_*Cd*_x_*Te and In*_x_*Ga_1−_*_x_*As/InP as a function of normalized intensity for all cases of Figure 16.

**Figure 19:**
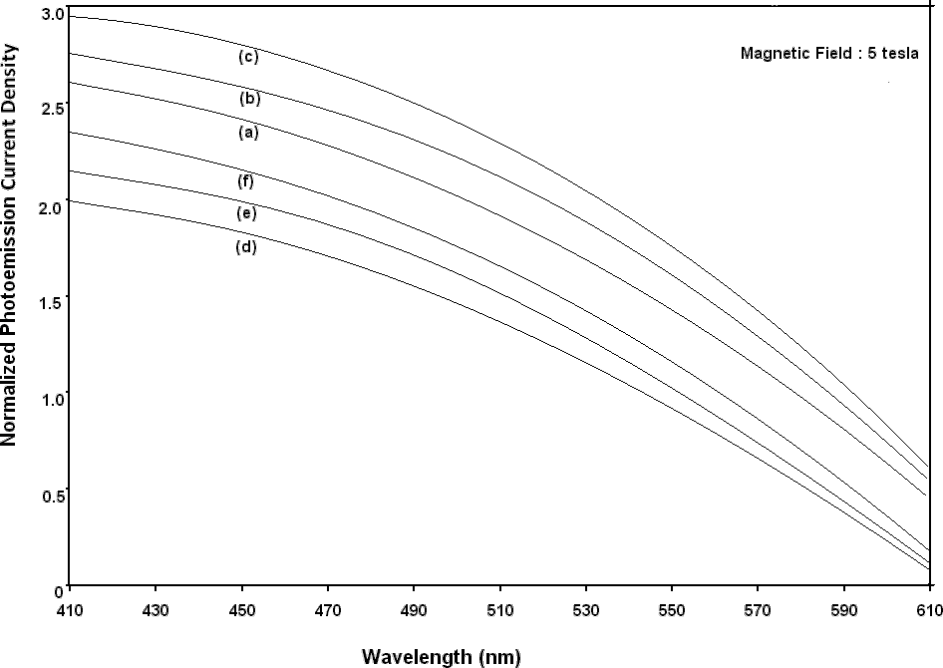
Plot of the normalized magneto photoemission current density from effective mass superlattices of HgTe/Hg_1−_*_x_*Cd*_x_*Te and In*_x_*Ga_1−_*_x_*As/InP as a function of wavelength for all cases of Figure 16.

**Figure 20:**
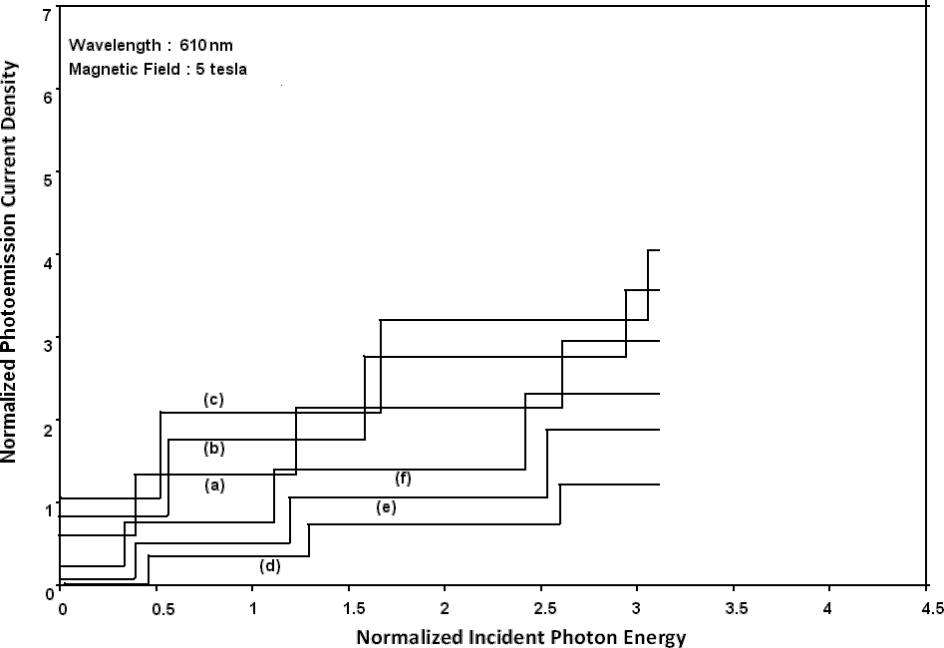
Plot of the normalized magneto photo-emitted current density from effective mass superlattices of HgTe/Hg_1−_*_x_*Cd*_x_*Te and In*_x_*Ga_1−_*_x_*As/InP as a function of normalized incident photon energy for all cases of Figure 16.

It appears from Figure 1 that the normalized photo-emitted current density from QW effective mass HgTe/Hg_1−_*_x_*Cd*_x_*Te and In*_x_*Ga_1−_*_x_*As/InP SLs oscillate with the inverse quantizing magnetic field due to the Shubnikov–de Haas (SdH) effect, where the oscillatory amplitudes and the numerical values are determined by the respective energy band constants. From Figure 2, it appears that the photo-emitted current density increases with increasing carrier concentration in an oscillatory way. Figure 3 and Figure 4 show that the photo-emitted current density decreases with increasing intensity and wavelength in different manners. From Figure 5, it appears that the normalized photo-emitted current density from QW effective mass HgTe/Hg_1−_*_x_*Cd*_x_*Te and In*_x_*Ga_1−_*_x_*As/InP SLs decreases with increasing film thickness in an oscillatory manner, with different numerical values as specified by the energy band constants of the aforementioned SLs. From Figure 6, it appears that the normalized photoemission from QWW effective mass HgTe/Hg_1−_*_x_*Cd*_x_*Te and In*_x_*Ga_1−_*_x_*As/InP SLs decreases with increasing thickness and exhibits large oscillations. From Figure 7, it appears that the normalized photocurrent for the said system increases with increasing carrier concentration, exhibiting a quantum jump for a particular value of the said variable, for all the models of both the SLs. From Figure 8 and Figure 9, it can be inferred that the normalized photocurrent in this case increases with decreasing intensity and wavelength in different manners. From Figure 10, it has been observed that the normalized photocurrent from QWW effective mass HgTe/Hg_1−_*_x_*Cd*_x_*Te and In*_x_*Ga_1−_*_x_*As/InP SLs increases with increasing normalized incident photon energy and exhibits quantum steps for specific values of the said variable.

From Figure 11, it appears that photo-emitted current density from QD effective mass HgTe/Hg_1−_*_x_*Cd*_x_*Te and In*_x_*Ga_1−_*_x_*As/InP SLs exhibits the same type of variations as given in Figure 5 and Figure 6, respectively, although the physics of QD effective mass SLs is completely different as compared to the magneto quantum well effective mass SLs and quantum wire effective mass SLs, respectively. The different physical phenomena in the QD case, as compared to the other two cases, exhibit quantum jumps at different numerical values of photoemission and different thicknesses, respectively. From Figure 12 to Figure 14, it appears that photoemission current density from QD effective mass HgTe/Hg_1−_*_x_*Cd*_x_*Te and In*_x_*Ga_1−_*_x_*As/InP SLs increases with increasing carrier concentration, decreasing intensity and decreasing wavelength, respectively, in various manners.

Figure 15 demonstrates the fact that the photoemission current density from QD effective mass HgTe/Hg_1−_*_x_*Cd*_x_*Te and In*_x_*Ga_1−_*_x_*As/InP SLs exhibits quantum steps with increasing photon energy for both the cases. Figure 16 exhibits the fact that the normalized photoemission current density from effective mass HgTe/Hg_1−_*_x_*Cd*_x_*Te and In*_x_*Ga_1−_*_x_*As/InP SLs oscillates with inverse quantizing magnetic field. Figure 17 exhibits the fact that the photoemission in this case increases with increasing carrier concentration. Figure 18 and Figure 19 demonstrate that photoemission current density decreases with increasing intensity and wavelength in different manners. Finally, from Figure 20, it can be inferred that photoemission exhibits step functional dependence with increasing photon energy for both the SLs, in this case with different numerical magnitudes.

The SL is a three dimensional system under periodic potential. The two dimensional dispersion relations of inversion layers of III–IV materials, for both weak and strong electric field limits, can be expressed as

[37]
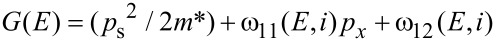


and

[38]
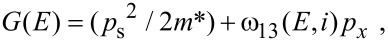


where the notations have been defined in [40]. The result for the low electric field limit cannot be at all connected with the corresponding results for the high electric limit in the analogous two-dimensional systems. There is a radical difference in the dispersion relation of the 3D quantized structures and the corresponding dispersion law of the 2D systems. From this paper, the dispersion relations of the various types of 1D system can be formulated and the corresponding photoemissions can also be investigated. The results will be fundamentally different in all cases and the reduction from lower to higher (or higher to lower) dimensions will not be possible due to system asymmetry, and they will reveal new physical features in the respective cases. The dispersion law and the corresponding wave function play a cardinal role in formulating any electronic property of any electronic material, since they change in a fundamental way when moving from one to three dimensions. Consequently all the formulations of the different transport quantities change radically.

It is imperative to state that our investigations excludes the many-body, hot electron, spin broadening, and the allied quantum dot and SL effects, in this simplified theoretical formalism, due to the absence of proper analytical techniques for including them in the generalized systems as considered here. Our simplified approach will be appropriate for the purpose of comparison when methods to tackle the formidable retrospective inclusion of the said effects for the generalized systems emerge. It is vital that the results of our simple theory get transformed to the well-known formulation of photoemission for wide-gap materials having parabolic energy bands. This indirect test will not only illustrate the mathematical compatibility of our formulation but will also show the fact that our simple analysis is a more generalized one, since one can obtain the corresponding results for materials having parabolic energy bands under certain limiting conditions from our present derivation. The experimental results for the verification of the theoretical analyses of this article are still not available in the literature. It is worth noting that our generalized formulation will be useful to analyze the experimental results when they materialize. The inclusion of the said effects would certainly increase the accuracy of the results, although the qualitative features of the photoemission would not change in the presence of the aforementioned effects.

Finally, it can be remarked that on the basis of the dispersion relations of the various quantized structures as discussed above, the plasma frequency, the heat capacity, the dia- and para-magnetic susceptibilities and the various important transport coefficients can be probed for all types of quantized structures as considered here. Thus our theoretical formulation comprises the dispersion relation dependent properties of various technologically important quantum-confined materials having different band-structures. We have not considered other types of compounds, in order to keep the presentation concise and succinct. With different sets of energy band parameters, we shall get different numerical values of the photoemission. The nature of variations of the photoemission, as shown here, would be similar for the other types of materials and the simplified analysis of this paper exhibits the basic qualitative features of the photoemission from such quantized structures. Finally, it may be noted that the basic aim of this paper is not solely to demonstrate the influence of quantum confinement on the photoemission from effective mass superlattices but also to formulate the appropriate carrier statistics in the most generalized form, since the transport and other phenomena in modern nanostructured devices having different band structures, and the derivation of the expressions of many important carrier properties, are based on the temperature-dependent carrier statistics in such materials.
